# Community-Level Procedure Volume and Patient Health Profiles Following PCI-Capable Facility Openings

**DOI:** 10.1001/jamanetworkopen.2026.2420

**Published:** 2026-03-30

**Authors:** Yu-Chu Shen, Nandita Sarkar, Renee Y. Hsia

**Affiliations:** 1 Department of Acquisitions, Finance, and Manpower, Naval Postgraduate School, Monterey, California; 2 National Bureau of Economic Research, Cambridge, Massachusetts; 3 Department of Emergency Medicine, University of California, San Francisco; 4 Philip R. Lee Institute for Health Policy Studies, University of California, San Francisco

## Abstract

**Importance:**

While the clinical benefits of timely percutaneous coronary intervention (PCI) are well established, it remains unclear whether the expansion of PCI-capable facilities enhances access to critical care or contributes to overuse.

**Objective:**

To assess whether new PCI-capable hospital openings are associated with changes in the overall procedural volume at the community level and the health characteristics of patients undergoing PCI.

**Design, Setting, and Participants:**

This cohort study used California all-payer data from January 1, 2011, to December 31, 2022, including 651 585 patients across 2348 communities (defined by zip code). Using a difference-in-differences framework, changes in PCI volume and patient characteristics in communities exposed to a PCI facility opening were compared with those without, stratified by baseline PCI access. Statistical analysis was completed between January and July 2025.

**Exposures:**

Opening of a PCI-capable facility within a 30-minute driving time of a zip code community.

**Main Outcomes and Measures:**

Community-level PCI volume and patient-level indicators, including primary diagnosis of stable angina, prior acute myocardial infarction (AMI) or coronary artery bypass grafting (CABG), and procedure complexity (number of vessels treated).

**Results:**

The final sample included 651 585 patients (463 526 male [71%]; 128 469 Hispanic [20%], 370 672 White [57%]); 47 003 patients (7%) lived in rural communities. At baseline, 84 349 patients (13%) had no access to PCI within 30 minutes. Community PCI volume increased by 7.5% (95% CI, 6.4%-8.6%) after a local PCI facility opened, with a 19.9% increase (95% CI, 15.7%-24.1%) in communities without prior 30-minute access. Among patients, the proportion with stable angina increased by 2.5 percentage points (95% CI, 2.0 to 3.1 percentage points), and by 3.5 percentage points (95% CI, 1.3 to 5.7 percentage points) in communities with no PCI at baseline. In communities with prior access, there was a 0.7 percentage point increase (95% CI, 0.3 to 1.1 percentage points) in patients without prior AMI or CABG and a 0.6 percentage point increase (95% CI, 0.4 to 0.9 percentage points) in those receiving PCI on 3 or more vessels. In contrast, communities with no baseline access to PCI experienced a 2.2 percentage point increase (95% CI, 0.3 to 4.1 percentage points) in single-vessel PCI and a 2.1 percentage point decrease (95% CI, −3.0 to −1.2 percentage points) in patients receiving PCI on 3 or more vessels.

**Conclusions and Relevance:**

In this retrospective cohort study of 651 585 patients, the opening of PCI-capable hospitals was associated with increased community PCI volumes, particularly in underserved areas. Changes in patient profiles suggested potential supply-induced demand in areas that had existing access, and a release of unmet need in previously underserved areas; these findings highlighted the dual implications of service expansion.

## Key Points

**Question:**

Does the opening of percutaneous coronary intervention (PCI)–capable facilities increase overall procedure volume and change the health profiles of patients receiving PCI?

**Findings:**

In this cohort study of 651 585 patients from 2348 California communities (2011 to 2022), community PCI volume increased by 7.5% after a new PCI-capable facility opened, and by nearly 20% in communities that previously lacked access. Increases in stable angina cases and divergent shifts in treatment complexity suggest the potential of both supply-induced demand where PCI was already available and release of pent-up demand where it was not.

**Meaning:**

These findings suggest that expanding PCI capacity may improve access in underserved areas and risk increasing low-value or discretionary use in communities where PCI services are already available.

## Teaser

This cohort study of patients in California examines how the opening of hospitals capable of percutaneous coronary intervention (PCI) may affect procedural volume and changes in health characteristics of patients undergoing the procedure.

## Introduction

Cardiovascular disease remains the leading cause of morbidity and mortality worldwide, contributing to almost 18 million deaths each year.^1^ While thrombolytic therapy is recommended when there is no timely access to percutaneous coronary intervention (PCI),^2^ PCI availability has expanded significantly over the past 2 decades.^3^ Timely access to PCI has played a crucial role in improving all-cause mortality by 16%, cardiovascular mortality by 31%, and subsequent myocardial infarction by 26% for patients with acute coronary syndromes (ACS),^4,5,6^ specifically in cases of ST-elevation myocardial infarction (STEMI).^4^

Despite these advancements, new centers are disproportionately opening in higher-resource areas.^3,7,8,9^ Some studies^10,11^ suggest that increased procedural capacity has contributed to the rise of discretionary PCI interventions performed in patients for whom medical management may be equally effective. This issue is particularly relevant for patients with stable angina, where guidelines recommend medical therapy as a first-line treatment,^12,13^ despite PCI being appropriate to treat stable angina in some situations. While in some cases PCI is appropriate to treat stable angina, studies^14,15^ have documented the overuse of PCI in this population. Additionally, the broader literature on procedural expansion in other fields suggests that financial incentives,^16,17^ hospital competition,^7,16^ and volume requirements^18^ may influence treatment patterns, potentially leading to changes in the patient populations undergoing PCI.

However, there is limited research on the connection between these 2 issues, and specifically how PCI expansion affects the overall volume, the types of procedures performed, and the characteristics of patients receiving PCI. The research objectives of this study are 2-fold: we sought to evaluate how the expansion of PCI centers was associated with (1) overall procedural volume at the community level and (2) changes health profiles and treatment received among patients who receive PCI. We also examined differences in these outcomes between communities with and without baseline PCI access, as prior research suggests that resource availability can shape treatment patterns.^19^ Given evidence that financial and competitive factors influence procedural growth, we hypothesized that increased PCI availability contributes to shifts in patient selection, potentially increasing use in patients without myocardial infarction or unstable angina in areas with existing access. Understanding these dynamics is critical to ensuring that PCI expansion improves equitable access to high-value care across communities rather than contributing to the increase of PCI among patients without ACS.

## Methods

This cohort study was approved by the institutional review board of the University of California, San Francisco, which did not require informed consent for the use of deidentified patient records. We followed the Strengthening the Reporting of Observational Studies in Epidemiology (STROBE) reporting guidelines.

### Data Sources and Study Population

We obtained patient-level data from 3 separate administrative databases provided by the California Department of Health Care Access and Information (HCAI): nonpublic patient discharge data (PDD), emergency department data (EDD), and ambulatory surgery data (ASD), allowing us to capture the complete PCI patient cohort in outpatient and inpatient settings. All 3 data sources include key variables, such as the patient’s residential zip code, procedure codes and date, primary diagnoses, demographics (eg, age, self-reported sex, race, and ethnicity), insurance status, and comorbid conditions. Race and ethnicity were included as covariates because prior research has demonstrated that they are associated with differences in cardiovascular disease presentation, treatment patterns, and clinical outcomes, making adjustment important to account for potential disparities. We derived hospital data from the annual HCAI facility utilization data, which contains critical variables such as total facility PCI procedure volume, ownership, and geographic information, including longitude and latitude coordinates. We acquired community data from the 2010 Census, which identified the longitude and latitude of the centroid of a given zip code community. In addition, we used web-based queries to develop a database that captures drive time between each community’s geographic center and any given facility, assuming normal traffic conditions.^20^ We restricted our analysis to California because it is one of the few states that maintains comprehensive, all-payer patient-level data across inpatient, emergency department, and ambulatory surgery encounters linked to annual facility-level utilization reports with PCI volumes and precise geographic coordinates.

Our study population included all patients, regardless of insurance coverage, in California who had received PCI any time between January 1, 2011, and December 31, 2022. Based on prior research and in consultation with cardiologists, we identified patients who received PCI based on the International Classification of Diseases procedure codes (*International Classification of Diseases, Ninth Revision [ICD-9]* before October 2015 and *International Statistical Classification of Diseases and Related Health Problems, Tenth Revision [ICD-10]* afterward). The complete list of codes is provided in eTable 1 in Supplement 1. We focused on PCI, rather than the broader definition sometimes used in studies that also include coronary angiography, because PCI more directly reflects clinical decision-making and resource allocation. We excluded patients without geographic information and those whose mailing zip code was more than 100 miles from the admitting facility because they likely received treatment away from home. Because this was a retrospective analysis of existing administrative data, there was no participant follow-up, and thus, loss to follow-up is not applicable. All eligible observations were drawn from complete encounter records within the study period.

### Identification of Hospital PCI Capability

To minimize self-reporting errors, we used a volume-based approach to determine whether and when a hospital acquired PCI technology.^9,19^ We defined a hospital as PCI capable if it performed at least 10 PCI procedures based on the 3 sources of patient discharge data (combined EDD, ASD, and PDD) in that year. In some instances where patient records were assigned to a parent facility, we used facility-level volume reports that could distinguish the subsidiaries or satellites from the parent to determine if the facility was PCI capable. In rare cases, if there was a data discrepancy, we hard-coded PCI capability after manually verifying the existence of the cardiac catheterization laboratory. To reduce measurement errors, we identified the opening year of the PCI facility as the first of 2 consecutive years in which the facility met the volume criteria. Our sensitivity analysis used a higher volume threshold of 50 procedures per year.

### Determining Community Access to PCI-Capable Hospitals

Starting in 2011, for each zip code community and each year, we first used the drive-time database to identify the number of PCI-capable facilities within a 30-minute drive time. Using 2011 as the baseline, we tracked PCI facility openings and closures between 2012 and 2022. A community was defined as exposed to a PCI opening on and after the year that a new PCI opened within 30 minutes of the community, regardless of whether there were preexisting PCI centers nearby. We use a 30-minute threshold as a widely applied benchmark for reasonable geographic access in health services research.^21,22,23,24,25^ This measure captures potential geographic access, not system time, which includes transport, emergency medical service activation, triage, and prehospital acquisition. Additionally, we chose a threshold of 15 minutes for an opening or closure based on studies^26,27^ showing that most hospital visits are within 15 minutes of where a patient resides.^28^ As explained in prior work,^19^ clinical data demonstrate that every 15-minute delay in receiving care after 90 minutes is associated with an increased risk of death for management of STEMI.^29^

### Community-Level Volume Determination

For our community-level analysis, we examined total community PCI volume when a community was exposed to a newly opened PCI lab within 30 minutes of drive time. Specifically, community PCI volume was defined as the total number of PCI procedures (combined from ambulatory, emergency, and inpatient encounters) performed on patients residing in a given community. Because self-reported PCI capability may not reflect actual procedural activity, and Medicare-based thresholds^19,30^ are not directly applicable to all-payer data, we used observed PCI volume to define PCI capability and assessed robustness across alternative thresholds. A community was defined as a zip code, the smallest geographic unit available in our data within the calendar year.

### Patient-Level Outcomes

We defined the health profile outcomes at the patient level. We examined 2 health profile proxies: whether the person’s primary diagnosis was stable angina, broadly defined as any admission that is not acute myocardial infarction or unstable angina,^31,32^ and whether the person had a prior incident of acute myocardial infarction or underwent CABG in the past 12 months. Because administrative data lack granular clinical markers of ischemia severity and symptom burden, we defined stable angina as nonacute coronary syndrome presentations (ie, encounters without a primary diagnosis of acute myocardial infarction or unstable angina), recognizing that this group is heterogeneous and includes patients with varying clinical indications for PCI. We hypothesize that PCI openings might lead to what may be described as “indication creep” and result in an increased proportion of healthier patients (ie, a primary diagnosis of stable angina or patients without prior acute heart conditions) who receive PCI as a share of total PCI volume. To further understand changes in patient types, we are also examining changes in the complexity of the procedure.^33,34,35^ Specifically, we looked at both ends of the distribution: the share of patients who had just 1 vessel treated during the PCI procedure (as a proxy for relatively simple, uncomplicated cases) and those who had procedures involving 3 or more vessels.

### Statistical Analysis

There are 2 parts to our analysis; both follow the difference-in-differences framework in that we compare changes in outcome in the treatment community (communities that experienced a PCI opening) before and after the PCI opening occurred relative to changes in outcome in the control community (communities that did not experience a PCI opening) during the same period. To address the first part of our research objective of determining how PCI facility openings affect total PCI volume, we conducted a community-level analysis using panel data where each observation represented a community-year. We implemented a community fixed-effects model where the dependent variable was the total community PCI volume as defined previously, which was log-transformed for more straightforward interpretation. The key independent variable was a binary variable that took on the value 1 on and after the year that a community experienced a PCI opening within a 30-minute driving time, and zero otherwise. Community fixed effects are critical because they control for unobserved differences across communities (such as underlying sickness and income distributions). In addition, we included the community’s demographics (share of female patients; Asian, Black, Hispanic, and other racial or ethnic groups [Native Hawaiian or other Pacific Islander, multiracial, other, unknown]; share of age groups) and insurance coverage (share of patients under Medicare, Medicaid, charity care, self-pay, and other coverage), and year dummies to capture macro trends. We first estimated the change in volume for all communities. Then we stratified the analysis by 3 groups based on the community’s baseline access to PCI: (1) access to a PCI-capable facility within 15 minutes, (2) no access to a PCI-capable facility within 15 minutes, and (3) no access to a PCI-capable facility within 30 minutes at baseline.

Then, we conducted a patient-level analysis to evaluate changes in the health profile of patients who received PCI before and after a community experienced PCI opening. Because all outcomes are binary, we implemented a linear probability model with community fixed effects to remove systematic differences between treatment and control communities and year fixed effects to control for macro trends common to all communities. The key independent variable was the same as the community-level analysis (ie, a postopening indicator). In all patient-level models, we controlled for demographic characteristics (sex, race and ethnicity, age groups, insurance coverage). To analyze the treatment received, we also controlled for primary diagnoses and comorbid conditions so that the coefficients represented changes in the probability of treatment between comparable patients with similar underlying health conditions in both the treatment and control communities.

Even though a probit or logit model is a natural choice for estimating a dichotomous variable in cross-sectional data, these models result in inconsistent estimators in panel data because we include many community fixed effects.^36,37^ The linear probability model can consistently estimate the impact of exposure to PCI opening on dichotomous outcomes.^38^ Based on results from the community-level analysis, we estimated the patient-level analysis separately for those residing in communities with a PCI facility within a 30-minute drive time at baseline and those in communities with no PCI access at baseline.

We conducted several sensitivity analyses to assess the robustness of our findings. First, we examined whether the effects differed by proximity, comparing PCI center openings within a 15-minute drive time vs those between 15 and 30 minutes. Second, we evaluated how outcomes varied depending on whether the opening represented the first PCI center within 30 minutes or a subsequent opening later in the study period. Lastly, we assessed differences based on the procedural volume of the newly opened PCI facilities, comparing low-volume facilities (10 to 50 annual cases) to higher-volume centers (50 annual cases or more). Statistical analysis was completed between January and July 2025 using Stata version 19 (StataCorp LLC). The threshold for significance was *P* < .05 in 2-sided tests.

## Results

Our final analytical file contains 651 585 patients from 2348 zip code communities; 70 580 patients (11%) identified as Asian, 33 668 Black (5%), 128 469 Hispanic (20%), 370 672 White (57%), and 46 504 (7%) with other racial and ethnic categories, according to race and ethnicity information in the administrative records (Table). In the full cohort, 567 236 patients (87%) had access to a PCI-capable facility within 30 minutes, while 84 349 (13%) did not during our study period.

**Table.** Descriptive Statistics of PCI Patient Characteristics by Baseline Access

Characteristics	Patients, No. (%)		
	Total (N = 651 585)	Had PCI within 30 min at baseline (n = 567 236)	No PCI within 30 min at baseline (n = 84 349)
No. of zip code communities	6819	741	6078
Community			
No PCI ≤30 min at baseline	84 349 (13)	NA	NA
Had PCI ≤30 min at baseline	567 236 (87)	NA	NA
Rural	47 003 (7)	11 523 (2)	35 480 (42)
Patient demographics			
Race or ethnicity			
American Indian	1692 (<1)	1305 (<1)	387 (<1)
Asian	70 580 (11)	68 098 (12)	387 (<1)
Black	33 668 (5)	31 612 (6)	2056 (2)
Hispanic	128 469 (20)	112 374 (20)	16 095 (19)
White	370 672 (57)	312 367 (55)	58 305 (69)
Other^a^	46 504 (7)	41 480 (7)	5024 (6)
Sex			
Female	188 059 (29)	162 586 (29)	25 473 (30)
Male	463 526 (71)	404 650 (71)	58 876 (70)
Age distribution at time of admission			
0-39 y	10 489 (2)	9299 (2)	1190 (1)
40-64 y	271 380 (42)	237 458 (42)	33 922 (40)
65-69 y	101 159 (16)	87 704 (15)	13 455 (16)
70-74 y	92 397 (14)	79 696 (14)	12 701 (15)
75-79 y	76 211 (12)	65 755 (12)	10 456 (12)
80-84 y	56 015 (9)	48 661 (9)	7354 (9)
≥85 y	43 863 (7)	38 599 (7)	5264 (6)
Insurance coverage			
Medicare	349 539 (54)	300 143 (53)	49 396 (59)
Medicaid or charity	83 915 (13)	73 637 (13)	10 278 (12)
Private	190 358 (29)	169 873 (30)	20 485 (24)
Self-pay	13 489 (2)	11 975 (2)	1514 (2)
Other	14 284 (2)	11 608 (2)	2676 (3)
Patient health profile			
Stable angina	350 281 (54)	304 849 (54)	45 432 (54)
STEMI	129 257 (20)	113 029 (20)	16 228 (19)
No prior acute heart condition	481 095 (74)	419 240 (74)	61 855 (73)
Comorbid conditions at time of admission			
Peripheral vascular disease	71 339 (11)	62 962 (11)	8377 (10)
Pulmonary circulation disorders	12 081 (2)	10 396 (2)	1685 (2)
Diabetes	230 957 (35)	202 117 (36)	28 840 (34)
Kidney failure	116 910 (18)	103 848 (18)	13 062 (15)
Liver	14 766 (2)	13 165 (2)	1601 (2)
Cancer	11 559 (2)	10 139 (2)	1420 (2)
Dementia	9358 (1)	8257 (1)	1101 (1)
Valvular disease	58 539 (9)	50 159 (9)	8380 (10)
Hypertension	495 985 (76)	431 121 (76)	64 864 (77)
Chronic pulmonary disease	87 431 (13)	74 256 (13)	13 175 (16)
Rheumatoid arthritis or collagen vascular	11 011 (2)	9616 (2)	1395 (2)
Coagulation deficiency	22 677 (3)	20 263 (4)	2414 (3)
Obesity	107 883 (17)	93 266 (16)	14 617 (17)
Psychosis	31 075 (5)	27 438 (5)	3637 (4)
Hypothyroidism	57 649 (9)	49 712 (9)	7937 (9)
Paralysis and other neurological disorder	25 885 (4)	22 656 (4)	3229 (4)
Ulcer	2050 (<1)	1784 (<1)	266 (<1)
Weight loss	10 130 (2)	9003 (2)	1127 (1)
Fluid and electrolyte disorders	83 805 (13)	73 844 (13)	9961 (12)
Anemia (blood loss and deficiency)	76 307 (12)	68 264 (12)	8043 (10)
Atrial flutter	88 723 (14)	76 698 (14)	12 025 (14)
Heart failure	159 424 (24)	138 736 (24)	20 688 (25)
Chronic kidney disease	123 246 (19)	109 504 (19)	13 742 (16)
Hyperlipidemia	459 250 (70)	400 698 (71)	58 552 (69)
Stroke	19 450 (3)	16 877 (3)	2573 (3)
Mental disorder	75 811 (12)	65 909 (12)	9902 (12)
Treatment received			
No. of vessels treated			
1	495 689 (76)	430 966 (76)	64 723 (77)
2	124 267 (19)	108 392 (19)	15 875 (19)
≥3	31 629 (5)	27 878 (5)	3751 (4)
Zip codes that experienced opening during study period	1211 (18)	40 (5)	1171 (19)
Patients who experienced opening during study period	460 202 (71)	17 565 (21)	442 637 (78)

Abbreviations: NA, not applicable; PCI, percutaneous coronary intervention; STEMI, ST-segment elevation myocardial infarction.

^a^ Includes Native Hawaiian or other Pacific Islander, multiracial, other, or unknown self-reported race.

**Figure 1.**
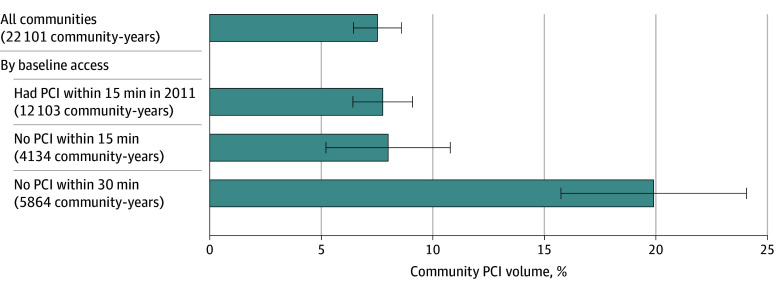
Bar Chart of Changes in Community Percutaneous Coronary Intervention (PCI) Volume On and After PCI Facility Opening Within 30 Minutes Between 2011 and 2022 Total unique communities represented included 1270 with baseline PCI access, 405 with no PCI within 15 minutes at baseline, and 673 with no PCI within 30 minutes at baseline. Dependent variable is log transformed PCI volume; community fixed effects model is weighted by community population size. Whiskers indicate 95% CIs.

Overall, total community PCI volume increased by 7.5% (95% CI, 6.4%-8.6%) on and after community exposure to a PCI opening within a 30-minute drive, relative to the control community (Figure 1; eTable 2 in Supplement 1).^39^ When stratifying by baseline access, communities with existing PCI centers within a 15-minute distance experienced a 7.8% increase (95% CI, 6.4%-9.1%) after an opening; communities that had no PCI within 15 minutes (but did have access within 30 minutes) also had a similar increase in volume, by 8.0% (95% CI, 5.2%-10.8%). In contrast, for communities without a PCI facility within 30 minutes, volume increased by 19.9% (95% CI, 15.7%-24.1%).

**Figure 2.**
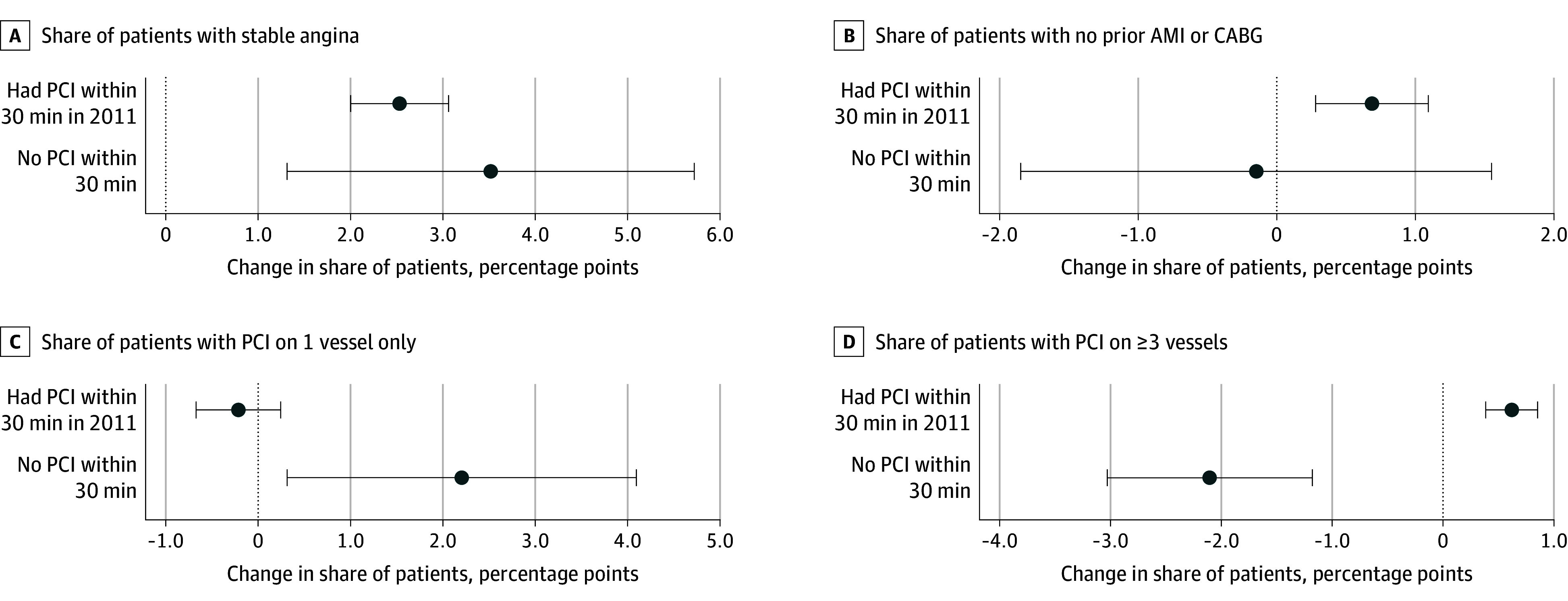
Dot Plot of Changes in Patient Health Profile and Case Complexity Measures After Percutaneous Coronary Intervention (PCI) Facility Opening Within a 30-Minute Drive There were 554 268 patients in communities with baseline PCI access in 2011, and 83 879 in communities with no PCI at baseline. Reference communities are those with the same baseline PCI status that did not experience a new PCI opening during the study period. Coefficients represent changes in percentage points, and error bars represent 95% CIs. AMI indicates acute myocardial infarction; CABG, coronary artery bypass grafting.

The introduction of new PCI facilities in communities was also associated with shifts in types of patients receiving PCI procedures. Due to substantial differences in the volume increase between communities with and without access to PCI within 30 minutes at baseline, we separately estimated all patient-level models for these 2 types of communities. Both types of markets experienced an increased share of patients with stable angina receiving PCI (Figure 2A; eTable 3 in Supplement 1). Specifically, in communities with baseline access, the share of PCI patients whose primary diagnosis was stable angina increased by 2.5 percentage points (95% CI, 2.0 to 3.1 percentage points). Given the mean rate of 54%, this represents a 4.7% relative increase. In communities with no baseline PCI access, the corresponding increase of patients with stable angina was 3.5 percentage points (95% CI, 1.3 to 5.7 percentage points), representing a 6.5% relative increase. In communities that already had PCI at baseline, the share of patients with no prior AMI or CABG increased slightly by 0.7 percentage points (95% CI, 0.3 to 1.1 percentage points), or a 0.9% relative increase, whereas there was no statistically significant change in communities with no PCI facility nearby at baseline.

In terms of treatment received, the share of patients who had revascularization of 1 vessel (as opposed to multiple vessels) stayed the same following PCI opening in communities that had PCI at baseline but increased by 2.2 percentage points (95% CI, 0.3 to 4.1 percentage points), or a 2.9% relative increase (given the mean rate of 77%) for communities that did not have PCI at baseline within 30 minutes (Figure 2C; eTable 4 in Supplement 1). The share of patients receiving PCI on 3 or more vessels increased in communities with baseline access by 0.6 percentage points (95% CI, 0.4 to 0.9 percentage points), a 12% relative increase (based off a mean base rate of 5%), and decreased by 2.1 percentage points (95% CI, −3.0 to −1.2 percentage points) in communities with no baseline access, a relative decrease of 42% (Figure 2D).

We performed the following sensitivity analyses on the patient-level analysis. First, we differentiated between openings that were within 15 minutes vs those that were between 15 and 30 minutes. We found that communities with no baseline access had similar results, except when evaluating the treatment of 1 vessel (eTable 5 in the Supplement). In this stratified analysis, openings that were within 15 minutes were associated with decreased probability of 1 vessel PCI in communities with baseline access and increased for communities without baseline access, while openings within 15 and 30 minutes were not associated with statistically significant changes in either group. Our second sensitivity analysis differentiated between the first opening and subsequent opening of PCI within 30 minutes. We found that changes in outcome were again similar, regardless of whether it was the first or subsequent opening in later years (eTable 6 in Supplement 1). Lastly, we differentiated between exposure to the opening of a very low-volume PCI facility (defined as 10 to 50 cases per year) and a higher-volume (50 or more cases) PCI facility. In communities with no baseline access, increases in the share of patients with stable angina and those with 1 vessel being treated were observed following higher-volume openings, whereas the share of patients with 3 or more vessels being treated was statistically significant for both volume types. The results for communities with existing access were similar, with increases in the share of patients with stable angina observed on and after a low-volume facility opening. For patients in this community with 1 vessel being treated, there was a statistically significant decrease observed in low-volume facilities, and statistically significant increases in both facility types for patients with 3 or more vessels being treated. In communities with existing PCI access, the increase in stable angina was concentrated among those exposed to very low volume (eTable 7 in Supplement 1). Treatment communities that experienced openings stratified by the year of opening are available in eTable 8 in Supplement 1.

## Discussion

In this cohort study of 651 585 patients who received PCI, we found that the introduction of a PCI-capable facility increased the overall volume of PCI done in a community by 7.5%. The rise in patient volume was substantially larger, increasing by almost 20%, in communities that had previously not had access to a PCI facility within a 30-minute drive. We also observed a notable shift in the case mix: after a PCI facility opened, there was an increase in the proportion of patients with stable angina receiving PCI in both communities that had baseline access to a PCI facility and communities that did not. In addition, we found that the types of patients receiving PCI differed between the 2 communities. In communities that already had access to PCI, the opening of a new PCI facility was associated with an increase in both less severely ill patients (those without a prior AMI or CABG) and more severely ill patients (those requiring PCI for 3 or more vessels). In communities with no baseline access, the share of patients without prior acute heart conditions remained similar, and the newly treated patients tended to receive PCI on 1 vessel only.

These findings demonstrate that the introduction of a new service line—even one like PCI, which is governed by well-established clinical guidelines—can meaningfully alter the volume and composition of care delivered at the community level. One possible explanation for this increase is pent-up demand, particularly in previously underserved areas.^8,40^ The nearly 20% increase in PCI volume in areas without prior access is consistent with a release of pent-up demand; however, the simultaneous increase in PCI among patients with stable angina across communities with or without access suggests that supply-driven increases in discretionary use may also be driving utilization. Together, these findings indicate that PCI expansion reflects both improved access and supply-induced demand.^9,41^ In these settings, PCI expansion may represent a move toward more equitable access to essential cardiac services.

However, the increases we observed may also reflect supplier-induced demand, a phenomenon in which the availability of a medical service influences its utilization, irrespective of changes in population health or clinical need.^42,43^ The increase in PCI among patients with stable angina^44,45^ in both communities with and without prior access raises concerns for overuse in lower-acuity patients, particularly given evidence that PCI does not offer significant benefits when measuring mortality, myocardial infarction, or other major cardiovascular events for patients with stable angina or significant coronary artery disease.^12,14,44^ It is also possible that competition between health care systems may incentivize higher procedural volume, especially in fee-for-service environments, where procedural interventions are financially rewarded. Previous research^19,46^ has shown that procedural growth can outpace clinical needs when new facilities are introduced, especially in high-revenue service lines like PCI.

Multivessel PCI is generally associated with a higher rate of urgent procedures, complex disease presentations,^33^ and higher in-hospital mortality rates.^34,35^ We therefore can interpret the rise in multivessel PCI as a share of all PCI in communities that already had baseline PCI access as a proxy for an increase in case severity. However, interpretation of multivessel PCI as a marker of case severity must be approached cautiously, as indications for PCI vs CABG in multivessel disease require equipoise and rely on anatomical and clinical factors not captured in administrative data. Shared decision-making and guideline-supported equipoise in certain scenarios cannot be assessed in our dataset. An opening of an additional PCI facility in these communities may have led patients who previously received alternative treatments, such as CABG, to now receive multivessel PCI. Given that studies have proven CABG to be superior to PCI for most patients with multivessel disease,^47^ this shift may also support the idea of supplier-induced demand, where the availability of a service can influence clinical decision-making. Overall, the fact that patients at both ends of the clinical severity spectrum are receiving more PCI after openings in communities with existing PCI access within 30 minutes suggests potential indication creep, where expanded capacity may lead to a broader interpretation of eligibility for certain procedures. In other words, health systems could be introducing new services to capture market share rather than to fill unmet clinical needs.^46^

### Limitations

This study has several limitations. First, our analysis used administrative data, which lacks clinical granularity. However, using alternative data sources, such as traditional registry data, misses hospitals that choose not to report their data and precludes population-level analysis to capture our outcomes. Second, there are inevitable measurement errors in defining hospital PCI capacity and calculating drive time. We believe these errors will not bias our findings, as we assume the errors are evenly distributed across both communities with and without PCI access. Third, the analysis was restricted to California data, which may limit the generalizability of our findings due to differences in state policies and geography. However, given that 1 in 8 US residents live in California,^48^ the state’s size and diversity may enhance the relevance of these findings to broader national trends. Fourth, while we used robust methods to assess changes associated with the opening of PCI-capable facilities, we cannot establish causality because our data do not show whether PCI facility openings increased the community volume or if PCI facilities were opened because of demand. Thus, unmeasured confounding factors, such as changes in referral patterns, physician behavior, or local health policy, may have contributed to increased PCI volume. Fifth, because we used linear probability models, a small share (5%) of observations (particularly for patients with no prior heart conditions) yielded predicted probabilities outside the 0 to 1 range, although this did not affect the estimation of average effects. Finally, while improved PCI access may influence downstream AMI outcomes and STEMI treatment strategies such as fibrinolysis use, these outcomes require a distinct analytic approach and are addressed in separate, complementary analyses.^41,49^

## Conclusions

In this retrospective cohort study of 651 585 patients included in our analysis, the introduction of PCI-capable facilities was associated with a statistically significant increase in procedure volume, up to 20% in communities that previously lacked access to PCI. Openings of PCI-capable facilities were also associated with an increased share of patients receiving PCI for stable angina. For communities without baseline access, openings were associated with an increased proportion of single-vessel interventions, suggesting a release of pent-up demand in patients who may have delayed care due to a prior lack of access to necessary procedures. Conversely, for communities with baseline access, openings of PCI-capable facilities appeared to increase the share of healthier patients (those without prior AMI and CABG) as well as patients with more severe disease (those requiring 3-vessel or more PCI).

## Notes

Supplement 1. eTable 1. Codes for identifying diagnostic PCI eTable 2. Complete regression results of Figure 1 eTable 3. Complete regression results of Figure 2A and 2B eTable 4. Complete regression results of Figure 2C and 2D eTable 5. Sensitivity analysis that separately models a PCI opening within 15-min and those between 15-30 min eTable 6. Sensitivity analysis that separately models the initial PCI opening and additional openings in subsequent years eTable 7. Sensitivity analysis that separately models the opening of a very low-volume PCI facility and other PCI facilities eTable 8. Number of treatment communities and patients by year of exposure to PCI opening

Supplement 2. Data Sharing Statement
