# Supplementary material for: Community-Level Procedure Volume and Patient Health Profiles Following PCI-Capable Facility Openings
Source: JAMA Netw Open. 2026 Mar 30;9(3):e262420. doi: 10.1001/jamanetworkopen.2026.2420 (PMC13036571; doi:10.1001/jamanetworkopen.2026.2420)
Supplement: Supplement 1. — eTable 1. Codes for identifying diagnostic PCI eTable 2. Complete regression results of Figure 1 eTable 3. Complete regression results of Figure 2A and 2B eTable 4. Complete regression results of Figure 2C and 2D eTable 5. Sensitivity analysis that separately models a PCI opening within 15-min and those between 15-30 min eTable 6. Sensitivity analysis that separately models the initial PCI opening and additional openings in subsequent years eTable 7. Sensitivity analysis that separately models the opening of a very low-volume PCI facility and other PCI facilities eTable 8. Number of treatment communities and patients by year of exposure to PCI opening [file jamanetwopen-e262420-s001.pdf]

Supplemental Online Content

Shen Y, Sarkar N, Hsia RY. Community-level procedure volume and patient health profiles following PCI-capable facility openings. *JAMA Netw Open.* 2026;9(3):e262420. doi:10.1001/jamanetworkopen.2026.2420

eTable 1. Codes for identifying diagnostic PCI

eTable 2. Complete regression results of Figure 1

eTable 3. Complete regression results of Figure 2A and 2B

eTable 4. Complete regression results of Figure 2C and 2D

eTable 5. Sensitivity analysis that separately models a PCI opening within 15-min and those between 15-30 min

eTable 6. Sensitivity analysis that separately models the initial PCI opening and additional openings in subsequent years

eTable 7. Sensitivity analysis that separately models the opening of a very low-volume PCI facility and other PCI facilities

eTable 8. Number of treatment communities and patients by year of exposure to PCI opening

This supplemental material has been provided by the authors to give readers additional information about their work.

© 2026 Shen Y et al. *JAMA Network Open*.

eTable 1. Codes for identifying diagnostic PCI

ICD-9	ICD-10 (for 027x3xx and 027x4xx codes, exclude xxxxZ6 and xxxxZZ)	CPT
0066	02703xx	92920
3601	02704xx	92921
3602	02713xx	92924
3605	02714xx	92925
3606	02723xx	92928
3607	02724xx	92929
	02733xx	92933
	02734xx	92934
	02C03Z6	92937
	02C03ZZ	92938
	02C04Z6	92941
	02C04ZZ	92943
	02C13Z6	92944
	02C13ZZ	92973
	02C14Z6	92975
	02C14ZZ	92978
	02C23Z6	92979
	02C23ZZ	93571
	02C24Z6	93572
	02C24ZZ	92980
	02C33Z6	92981
	02C33ZZ	92982
	02C34Z6	92984
	02C34ZZ	92995
	02H03DZ	92996
	02H03YZ	
	02H13DZ	
	02H13YZ	
	02H23DZ	
	02H23YZ	
	02H33DZ	
	02H33YZ	
	X2C0361	
	X2C1361	
	X2C2361	
	X2C3361	

ICD = International Classification of Diseases
CPT= Current Procedural Terminology

© 2026 Shen Y et al. *JAMA Network Open*.

eTable 2. Complete regression results of Figure 1

Coefficient [95% CI]	All communities	By Baseline Access: Had PCI within 15-minute	no PCI within 15-minute	no PCI within 30-minute
On and after PCI opening within 30-min drive time	0.075**	0.078**	0.080**	0.199**
	[0.064,0.086]	[0.064,0.091]	[0.052,0.108]	[0.157,0.241]
**Mean community demographic characteristics**				
Female	0.009	-0.008	0.065	-0.000
	[-0.022,0.040]	[-0.052,0.036]	[-0.008,0.138]	[-0.052,0.052]
Black	-0.028	-0.044	-0.075	-0.019
	[-0.099,0.043]	[-0.130,0.043]	[-0.259,0.109]	[-0.207,0.169]
Hispanic	-0.022	-0.024	-0.030	-0.036
	[-0.063,0.018]	[-0.079,0.032]	[-0.123,0.063]	[-0.110,0.037]
Asian	0.019	-0.005	0.081	0.154
	[-0.036,0.075]	[-0.071,0.060]	[-0.066,0.228]	[-0.039,0.346]
Other races	-0.071*	-0.071	0.038	-0.152*
	[-0.130,-0.011]	[-0.150,0.008]	[-0.104,0.180]	[-0.270,-0.034]
65-69 years	0.060*	0.051	0.037	0.101*
	[0.014,0.107]	[-0.013,0.115]	[-0.072,0.147]	[0.020,0.182]
70-74 years	0.030	0.006	0.013	0.095*
	[-0.019,0.080]	[-0.063,0.074]	[-0.105,0.131]	[0.009,0.181]
75-79 years	0.092**	0.066	0.037	0.168**
	[0.038,0.145]	[-0.010,0.141]	[-0.089,0.164]	[0.078,0.258]
80-84 years	0.094**	0.104*	0.071	0.081
	[0.033,0.154]	[0.020,0.187]	[-0.072,0.214]	[-0.024,0.187]
85+ years	0.034	0.037	-0.013	0.035
	[-0.032,0.100]	[-0.054,0.128]	[-0.173,0.147]	[-0.083,0.153]
**Mean community insurance coverage**				
Medicare	0.085**	0.157**	-0.036	0.011
	[0.044,0.127]	[0.101,0.213]	[-0.136,0.064]	[-0.064,0.086]
Medicaid	0.010	0.067*	-0.087	-0.075
	[-0.037,0.057]	[0.002,0.132]	[-0.203,0.029]	[-0.157,0.008]
indigent	0.191**	0.096	0.245	0.338*
	[0.054,0.327]	[-0.082,0.273]	[-0.115,0.604]	[0.071,0.605]
Self-pay	-0.172**	-0.318**	0.225	-0.117
	[-0.267,-0.078]	[-0.449,-0.187]	[-0.012,0.462]	[-0.280,0.046]
Other coverage	0.074	0.111	-0.011	0.024
	[-0.013,0.160]	[-0.020,0.241]	[-0.212,0.190]	[-0.111,0.160]

© 2026 Shen Y et al. *JAMA Network Open*.

**Year trend (2011 is reference)**				
2012	-0.060**	-0.068**	-0.023	-0.081**
	[-0.074,-0.047]	[-0.085,-0.051]	[-0.056,0.010]	[-0.112,-0.049]
2013	-0.115**	-0.129**	-0.071**	-0.121**
	[-0.129,-0.102]	[-0.146,-0.112]	[-0.104,-0.038]	[-0.153,-0.090]
2014	-0.097**	-0.122**	-0.019	-0.092**
	[-0.111,-0.082]	[-0.140,-0.104]	[-0.054,0.016]	[-0.125,-0.060]
2015	-0.056**	-0.076**	0.016	-0.084**
	[-0.072,-0.039]	[-0.097,-0.055]	[-0.023,0.055]	[-0.120,-0.048]
2016	-0.043**	-0.069**	0.047	-0.054
	[-0.069,-0.017]	[-0.102,-0.037]	[-0.019,0.113]	[-0.116,0.007]
2017	-0.025	-0.056**	0.065	-0.014
	[-0.051,0.002]	[-0.089,-0.024]	[-0.001,0.131]	[-0.075,0.048]
2018	-0.037**	-0.069**	0.050	-0.023
	[-0.063,-0.011]	[-0.101,-0.036]	[-0.016,0.116]	[-0.085,0.039]
2019	0.001	-0.029	0.074*	0.023
	[-0.025,0.027]	[-0.062,0.004]	[0.007,0.140]	[-0.039,0.084]
2020	-0.145**	-0.188**	-0.029	-0.108**
	[-0.171,-0.119]	[-0.221,-0.155]	[-0.095,0.037]	[-0.170,-0.047]
2021	-0.070**	-0.094**	0.015	-0.093**
	[-0.097,-0.044]	[-0.128,-0.061]	[-0.051,0.082]	[-0.155,-0.031]
2022	-0.096**	-0.125**	0.002	-0.112**
	[-0.122,-0.069]	[-0.158,-0.091]	[-0.065,0.069]	[-0.175,-0.050]
ICD-10 period (post Oct 2015)	0.030**	0.029*	0.020	0.040
	[0.009,0.051]	[0.003,0.055]	[-0.035,0.075]	[-0.012,0.092]
Constant	3.827**	3.919**	3.713**	3.458**
	[3.800,3.854]	[3.882,3.957]	[3.652,3.773]	[3.407,3.509]
N	22101	12103	4134	5864

Note: Dependent variables are log transformed, and coefficients represent relative changes in percent (0.075 = 7.5%), and 95% CI are reported in brackets. Reference communities are those with the same baseline PCI status but did not experience new PCI openings during the study period. Reference group for race=white, for age = under 65, and for insurance = private insurance. ** p<0.01 * p<0.05

© 2026 Shen Y et al. *JAMA Network Open*.

eTable 3. Complete regression results of Figure 2A and 2B

	Stable angina		No prior acute heart condition	
	Had PCI within 30-min	No PCI at baseline	Had PCI within 30-min	No PCI at baseline
On and after PCI opening within 30-min drive time	2.53**	3.52**	0.69**	-0.15
	[2.00,3.06]	[1.31,5.72]	[0.28,1.09]	[-1.85,1.55]
**Patient characteristics**				
Female	-1.47**	-0.44	-1.66**	-1.02**
	[-1.76,-1.18]	[-1.17,0.29]	[-1.88,-1.43]	[-1.59,-0.46]
African-American	-2.13**	-1.42	-2.32**	-0.85
	[-2.76,-1.49]	[-3.63,0.80]	[-2.81,-1.84]	[-2.55,0.86]
Hispanic	-2.55**	-0.45	-1.53**	-1.57**
	[-2.93,-2.16]	[-1.43,0.54]	[-1.82,-1.24]	[-2.33,-0.81]
Asian or Pacific Islander	1.38**	1.56	-1.45**	-2.39**
	[0.93,1.83]	[-0.45,3.58]	[-1.79,-1.11]	[-3.94,-0.84]
Race other than above; may be mixed	-0.69*	-4.95**	-0.13	-1.44*
	[-1.25,-0.13]	[-6.68,-3.23]	[-0.56,0.30]	[-2.77,-0.11]
age group 65-69	5.46**	6.11**	1.07**	1.91**
	[5.01,5.90]	[4.94,7.28]	[0.73,1.42]	[1.00,2.81]
age group 70-74	6.60**	7.54**	0.92**	1.34**
	[6.12,7.08]	[6.30,8.78]	[0.55,1.29]	[0.38,2.29]
age group 75-79	6.30**	9.07**	-0.04	2.00**
	[5.78,6.81]	[7.75,10.39]	[-0.44,0.35]	[0.98,3.02]
age group 80-84	3.62**	5.63**	-1.27**	-0.24
	[3.05,4.19]	[4.17,7.09]	[-1.71,-0.84]	[-1.37,0.89]
age group 85	-3.35**	-0.68	-3.04**	-1.62*
	[-3.97,-2.74]	[-2.31,0.95]	[-3.52,-2.57]	[-2.88,-0.36]
**Insurance coverage (ref is private insurance)**				
Expected source of payment: Medicare	5.51**	3.02**	-3.39**	-3.30**
	[5.10,5.91]	[1.94,4.11]	[-3.70,-3.08]	[-4.14,-2.46]
Expected source of payment: Medicaid	-3.86**	-5.86**	-2.47**	-3.21**
	[-4.31,-3.40]	[-7.08,-4.64]	[-2.82,-2.13]	[-4.15,-2.27]
Indigent: county or other	-13.99**	-17.08**	-1.05	-1.83

© 2026 Shen Y et al. *JAMA Network Open*.

	[-15.44,-12.54]	[-21.05,-13.11]	[-2.16,0.06]	[-4.89,1.24]
Expected source of payment: Patient	-21.88**	-23.68**	1.32**	-0.44
	[-22.82,-20.94]	[-26.25,-21.10]	[0.60,2.05]	[-2.44,1.55]
Expected source of payment: Other than above	-3.68**	-2.44*	-1.32**	-0.66
	[-4.63,-2.73]	[-4.45,-0.43]	[-2.05,-0.59]	[-2.21,0.89]
Patient has no linkage identifier	-10.02**	-10.68**	28.32**	31.06**
	[-10.49,-9.55]	[-11.90,-9.45]	[27.96,28.68]	[30.11,32.01]
**Time trends**				
2011 (reference)	0.00	0.00	0.00	0.00
	[0.00,0.00]	[0.00,0.00]	[0.00,0.00]	[0.00,0.00]
2012	-2.91**	-3.32**	-2.28**	-3.44**
	[-3.56,-2.25]	[-4.96,-1.68]	[-2.79,-1.77]	[-4.71,-2.18]
2013	-5.77**	-6.15**	-2.12**	-3.27**
	[-6.45,-5.10]	[-7.81,-4.49]	[-2.63,-1.60]	[-4.55,-1.98]
2014	-6.47**	-6.31**	-2.09**	-3.04**
	[-7.17,-5.77]	[-7.97,-4.65]	[-2.63,-1.55]	[-4.32,-1.76]
2015	-6.82**	-6.78**	-2.63**	-2.57**
	[-7.60,-6.04]	[-8.57,-4.99]	[-3.23,-2.03]	[-3.95,-1.18]
2016	-6.09**	-2.89	-2.25**	-1.55
	[-7.35,-4.83]	[-6.07,0.28]	[-3.21,-1.28]	[-4.00,0.90]
2017	-6.62**	-3.07	-1.99**	-1.33
	[-7.88,-5.36]	[-6.24,0.10]	[-2.96,-1.02]	[-3.78,1.12]
2018	-6.98**	-4.36**	-2.31**	-1.83
	[-8.24,-5.71]	[-7.54,-1.19]	[-3.28,-1.34]	[-4.27,0.62]
2019	-6.23**	-2.86	-1.83**	-1.92
	[-7.50,-4.97]	[-6.02,0.31]	[-2.80,-0.86]	[-4.36,0.52]
2020	-6.99**	-3.70*	-2.01**	-2.14
	[-8.27,-5.72]	[-6.90,-0.51]	[-2.99,-1.03]	[-4.61,0.32]
2021	-5.58**	-1.93	-2.20**	-2.49*
	[-6.86,-4.31]	[-5.12,1.26]	[-3.18,-1.22]	[-4.95,-0.03]
2022	-5.56**	-3.41*	-2.84**	-2.23
	[-6.84,-4.29]	[-6.61,-0.21]	[-3.82,-1.86]	[-4.70,0.24]
On and after switch to ICD-10 coding (Oct 2015)	0.14	-3.24*	-1.30**	-3.63**
	[-0.88,1.17]	[-5.96,-0.52]	[-2.08,-0.51]	[-5.73,-1.54]

© 2026 Shen Y et al. *JAMA Network Open*.

**Primary diagnosis**				
Non-ST-elevation myocardial infarction (reference)			0.00<br>[0.00,0.00]	0.00<br>[0.00,0.00]
ST-segment elevation myocardial infarction			51.38**<br>[49.50,53.26]	53.98**<br>[48.60,59.37]
Unstable angina			50.08**<br>[49.83,50.33]	50.83**<br>[50.18,51.47]
Ischemic heart disease			33.63**<br>[33.29,33.96]	37.21**<br>[36.33,38.08]
Other			47.34**<br>[47.05,47.64]	45.21**<br>[44.44,45.98]
_cons	55.21**<br>[54.71,55.72]	56.10**<br>[54.78,57.41]	42.59**<br>[42.17,43.02]	43.08**<br>[41.96,44.19]
N	554,268	83,879	554,268	83,879

Note: Based on linear probability model with community fixed effects. Reference communities are those with the same baseline PCI status but did not experience new PCI opening during the study period. Reference group for race=white, for age = under 65, and for insurance = private insurance. Coefficients represent changes in percentage points, and numbers inside brackets represent 95% CI. ** p<0.01 * p<0.05

© 2026 Shen Y et al. *JAMA Network Open*.

eTable 4. Complete regression results of Figure 2C and 2D

	1 vessel		3 vessels	
	**Had PCI within 30-min**	**No PCI at baseline**	**Had PCI within 30-min**	**No PCI at baseline**
On and after PCI opening within 30-min drive time	-0.21	2.20*	0.62**	-2.10**
	[-0.67,0.25]	[0.31,4.09]	[0.39,0.85]	[-3.03,-1.18]
**Patient characteristics**				
Female	2.19**	2.18**	-0.81**	-0.68**
	[1.93,2.45]	[1.53,2.82]	[-0.94,-0.67]	[-1.00,-0.36]
African-American	2.33**	1.81	-0.81**	-1.05*
	[1.78,2.88]	[-0.09,3.71]	[-1.09,-0.53]	[-1.98,-0.11]
Hispanic	0.15	0.14	-0.17	-0.32
	[-0.18,0.48]	[-0.72,0.99]	[-0.34,0.00]	[-0.74,0.09]
Asian or Pacific Islander	-0.68**	-2.29**	0.05	0.75
	[-1.07,-0.29]	[-4.02,-0.55]	[-0.15,0.25]	[-0.10,1.60]
Race other than above; may be mixed	-0.57*	-2.17**	0.07	0.74*
	[-1.06,-0.09]	[-3.65,-0.68]	[-0.18,0.32]	[0.02,1.47]
age group 65-69	-0.78**	-0.73	0.03	-0.25
	[-1.17,-0.39]	[-1.74,0.28]	[-0.17,0.23]	[-0.75,0.24]
age group 70-74	-0.17	-0.59	-0.28*	-0.33
	[-0.59,0.25]	[-1.66,0.48]	[-0.49,-0.06]	[-0.85,0.20]
age group 75-79	0.01	-0.29	-0.29*	-0.10
	[-0.44,0.47]	[-1.43,0.86]	[-0.52,-0.06]	[-0.66,0.46]
age group 80-84	0.03	-1.93**	-0.28*	0.22
	[-0.47,0.53]	[-3.21,-0.66]	[-0.54,-0.03]	[-0.41,0.85]
age group 85+	0.06	0.05	-0.32*	0.23
	[-0.49,0.61]	[-1.40,1.49]	[-0.60,-0.04]	[-0.48,0.93]

© 2026 Shen Y et al. *JAMA Network Open*.

**Insurance coverage**				
Expected source of payment: Medicare	0.90**	0.87	-0.14	-0.42
	[0.54,1.25]	[-0.07,1.82]	[-0.33,0.04]	[-0.88,0.04]
Expected source of payment: Medicaid	1.91**	0.97	-0.65**	-0.33
	[1.51,2.31]	[-0.08,2.03]	[-0.85,-0.44]	[-0.85,0.19]
Indigent: county or other	2.91**	3.05	-1.10**	-0.80
	[1.65,4.16]	[-0.36,6.46]	[-1.74,-0.46]	[-2.47,0.87]
Expected source of payment: Patient	1.53**	1.72	-0.69**	-0.25
	[0.71,2.35]	[-0.50,3.94]	[-1.11,-0.27]	[-1.34,0.83]
Expected source of payment: Other than above	1.58**	2.29**	-0.69**	-0.36
	[0.76,2.41]	[0.56,4.01]	[-1.11,-0.27]	[-1.20,0.49]
Patient has no linkage identifier	-1.23**	-1.04	0.61**	-0.15
	[-1.64,-0.82]	[-2.09,0.02]	[0.40,0.82]	[-0.67,0.37]
**Time trends**				
2011	0.00	0.00	0.00	0.00
	[0.00,0.00]	[0.00,0.00]	[0.00,0.00]	[0.00,0.00]
2012	0.14	-0.38	-0.25	0.21
	[-0.43,0.71]	[-1.79,1.03]	[-0.54,0.04]	[-0.48,0.90]
2013	-1.02**	-2.24**	0.56**	0.77*
	[-1.60,-0.43]	[-3.67,-0.81]	[0.26,0.86]	[0.07,1.47]
2014	-2.26**	-2.05**	0.92**	0.88*
	[-2.87,-1.66]	[-3.48,-0.63]	[0.61,1.23]	[0.18,1.57]
2015	-2.50**	-3.14**	0.76**	1.12**
	[-3.18,-1.83]	[-4.67,-1.60]	[0.41,1.10]	[0.37,1.87]
2016	-0.81	-0.70	-0.07	-1.01
	[-1.91,0.28]	[-3.42,2.02]	[-0.63,0.49]	[-2.34,0.33]
2017	1.32*	1.15	-1.12**	-1.55*
	[0.23,2.41]	[-1.57,3.88]	[-1.68,-0.57]	[-2.88,-0.21]

© 2026 Shen Y et al. *JAMA Network Open*.

2018	0.81	0.26	-0.87**	-1.25
	[-0.28,1.90]	[-2.47,2.98]	[-1.43,-0.31]	[-2.58,0.09]
2019	0.31	0.18	-0.73*	-0.88
	[-0.79,1.40]	[-2.53,2.90]	[-1.28,-0.17]	[-2.21,0.45]
2020	-0.74	-1.15	-0.36	-0.27
	[-1.85,0.37]	[-3.89,1.59]	[-0.92,0.20]	[-1.61,1.08]
2021	-1.56**	-3.11*	0.19	-0.04
	[-2.67,-0.46]	[-5.85,-0.37]	[-0.38,0.75]	[-1.39,1.30]
2022	-1.02	-2.41	0.50	0.54
	[-2.12,0.09]	[-5.16,0.34]	[-0.07,1.06]	[-0.81,1.89]
On and after switch to ICD-10 coding (Oct 2015)	-2.31**	-3.07**	1.25**	2.52**
	[-3.20,-1.43]	[-5.41,-0.74]	[0.80,1.70]	[1.37,3.66]
**Patient comorbidities**				
Peripheral vascular disease	-0.81**	-1.51**	0.58**	0.11
	[-1.17,-0.44]	[-2.48,-0.53]	[0.39,0.77]	[-0.37,0.59]
Pulmonary Circulation disorders	-0.24	1.08	0.28	-0.54
	[-1.09,0.60]	[-1.00,3.16]	[-0.15,0.71]	[-1.56,0.48]
Diabetes (uncomp+complicated)	-1.03**	-0.72*	0.24**	0.33*
	[-1.28,-0.79]	[-1.35,-0.09]	[0.12,0.37]	[0.02,0.63]
Renal failure	-1.58**	-2.17	1.12**	0.61
	[-2.47,-0.69]	[-4.64,0.31]	[0.67,1.58]	[-0.60,1.82]
Liver disease	0.15	0.06	-0.06	-0.05
	[-0.59,0.90]	[-2.04,2.16]	[-0.44,0.32]	[-1.08,0.98]
Cancer	0.59	2.75*	0.29	-0.82
	[-0.25,1.43]	[0.53,4.97]	[-0.14,0.72]	[-1.91,0.26]
Dementia	0.30	-0.41	-0.08	0.47
	[-0.66,1.26]	[-2.97,2.15]	[-0.57,0.41]	[-0.79,1.72]
Valvular disease	-0.64**	-1.05*	0.11	0.36

© 2026 Shen Y et al. *JAMA Network Open*.

	[-1.04,-0.23]	[-2.03,-0.06]	[-0.09,0.32]	[-0.13,0.84]
Hypertension (uncomp+complicated)	-0.33*	-0.32	-0.04	-0.09
	[-0.60,-0.05]	[-1.03,0.38]	[-0.18,0.10]	[-0.43,0.26]
Chronic pulmonary disease	1.16**	0.50	-0.41**	-0.31
	[0.82,1.50]	[-0.31,1.32]	[-0.59,-0.24]	[-0.70,0.09]
Rheumatoid arthritis/collagen vascular	-0.27	1.02	-0.16	-0.36
	[-1.13,0.60]	[-1.21,3.26]	[-0.60,0.28]	[-1.46,0.73]
Coagulation deficiency	-0.71*	-1.33	0.68**	1.11*
	[-1.32,-0.09]	[-3.08,0.42]	[0.37,1.00]	[0.25,1.97]
Obesity	0.66**	1.32**	-0.33**	-0.49*
	[0.35,0.97]	[0.54,2.09]	[-0.49,-0.17]	[-0.87,-0.11]
Substance abuse	2.63**	2.85**	-0.89**	-1.14**
	[2.07,3.19]	[1.52,4.18]	[-1.18,-0.61]	[-1.79,-0.48]
Depression	1.07**	0.72	-0.57**	0.38
	[0.41,1.73]	[-1.00,2.44]	[-0.90,-0.23]	[-0.46,1.23]
Psychosis	0.26	-0.13	-0.11	-0.42
	[-0.36,0.88]	[-1.89,1.63]	[-0.43,0.21]	[-1.28,0.44]
Hypothyroidism	0.35	0.73	-0.01	-0.53*
	[-0.06,0.75]	[-0.27,1.74]	[-0.21,0.20]	[-1.02,-0.04]
Paralysis and other neurological disorder	0.57	-1.00	-0.16	-0.11
	[-0.02,1.15]	[-2.52,0.52]	[-0.45,0.14]	[-0.85,0.63]
Chronic Peptic ulcer disease	2.10*	-1.47	-0.21	0.75
	[0.12,4.09]	[-6.54,3.60]	[-1.22,0.80]	[-1.73,3.24]
Weight loss	-0.66	-1.66	0.02	0.70
	[-1.58,0.25]	[-4.18,0.87]	[-0.45,0.48]	[-0.54,1.94]
Fluid and electrolyte disorders	-0.07	-0.03	-0.03	0.12
	[-0.43,0.29]	[-1.00,0.93]	[-0.21,0.16]	[-0.35,0.59]
Anemia (blood loss and deficiency)	-0.98**	-1.08*	0.36**	0.21
	[-1.35,-0.60]	[-2.15,-0.01]	[0.17,0.55]	[-0.31,0.74]

© 2026 Shen Y et al. *JAMA Network Open*.

Stroke	-2.22**	-1.97*	0.92**	2.23**
	[-2.88,-1.56]	[-3.66,-0.29]	[0.58,1.25]	[1.41,3.05]
Chronic Kidney Disease	1.13*	0.97	-0.81**	-0.23
	[0.25,2.01]	[-1.47,3.41]	[-1.26,-0.36]	[-1.43,0.96]
Heart failure	-2.35**	-2.16**	0.97**	0.90**
	[-2.64,-2.06]	[-2.90,-1.43]	[0.82,1.12]	[0.54,1.26]
**Primary diagnosis**				
Non-ST-elevation myocardial infarction	0.00	0.00	0.00	0.00
	[0.00,0.00]	[0.00,0.00]	[0.00,0.00]	[0.00,0.00]
ST-segment elevation myocardial infarction	0.49	-4.09	0.15	0.15
	[-1.64,2.61]	[-10.09,1.90]	[-0.93,1.23]	[-2.79,3.08]
Unstable angina	-5.53**	-3.65**	2.28**	1.45**
	[-5.82,-5.24]	[-4.38,-2.92]	[2.14,2.43]	[1.09,1.81]
Ischemic heart disease	0.86**	0.27	-0.41**	-0.15
	[0.48,1.24]	[-0.71,1.25]	[-0.60,-0.21]	[-0.63,0.33]
Other	9.18**	8.01**	-2.29**	-2.04**
	[8.85,9.52]	[7.14,8.87]	[-2.46,-2.12]	[-2.46,-1.62]
_cons	78.36**	79.60**	3.62**	3.55**
	[77.83,78.88]	[78.25,80.96]	[3.35,3.88]	[2.89,4.22]
N	554,268	83,879	554,268	83,879

Note: Based on linear probability model with community fixed effects. Reference communities are those with the same baseline PCI status but did not experience new PCI opening during the study period. Reference group for race=white, for age = under 65, and for insurance = private insurance. Coefficients represent absolute changes in percentage points, and numbers inside brackets represent 95% CI. ** p<0.01 * p<0.05

© 2026 Shen Y et al. *JAMA Network Open*.

eTable 5. Sensitivity analysis that separately models a PCI opening within 15 min and those between 15-30 min

	Stable angina		No prior acute heart condition		1 vessel		3+ vessels	
	Had PCI within 30-min	No PCI at baseline	Had PCI within 30-min	No PCI at baseline	Had PCI within 30-min	No PCI at baseline	Had PCI within 30-min	No PCI at baseline
On and after PCI opening within 15-min drive time	2.52**	4.13*	0.65*	-1.97	-0.90**	3.57*	0.96**	-2.10**
	[1.85,3.18]	[0.71,7.55]	[0.13,1.17]	[-4.66,0.73]	[-1.47,-0.32]	[0.64,6.50]	[0.67,1.26]	[-3.54,-0.66]
On and after PCI opening within 30-min drive time	2.55**	3.38**	0.60**	0.13	0.07	1.71	0.48**	-2.16**
	[2.00,3.10]	[0.92,5.84]	[0.18,1.03]	[-1.81,2.07]	[-0.41,0.54]	[-0.39,3.82]	[0.23,0.72]	[-3.19,-1.12]

Note: Based on linear probability model with community fixed effects. Reference communities are those with the same baseline PCI status but did not experience new PCI opening during the study period. Coefficients represent changes in percentage points, and numbers inside brackets represent 95% CI. ** p<0.01 * p<0.05

© 2026 Shen Y et al. *JAMA Network Open*.

eTable 6. Sensitivity analysis that separately models initial PCI opening and additional openings in subsequent years

	Stable angina		No prior acute heart condition		1 vessel		3+ vessels	
	**Had PCI within 30-min**	**No PCI at baseline**	**Had PCI within 30-min**	**No PCI at baseline**	**Had PCI within 30-min**	**No PCI at baseline**	**Had PCI within 30-min**	**No PCI at baseline**
On and after first PCI opening within 30-min drive time	2.54** [2.01,3.07]	3.31** [0.99,5.62]	0.62** [0.20,1.03]	0.13 [-1.70,1.95]	-0.19 [-0.64,0.27]	2.05* [0.07,4.04]	0.60** [0.37,0.84]	-2.12** [-3.09,-1.14]
On and after subsequent PCI opening within 30-min drive time	2.45** [1.71,3.19]	4.34** [1.52,7.15]	0.97** [0.40,1.55]	-2.00 [-4.22,0.22]	-0.53 [-1.17,0.11]	2.77* [0.36,5.18]	0.65** [0.32,0.97]	-2.20** [-3.38,-1.01]

Note: Based on linear probability model with community fixed effects. Reference period for the treatment community is the pre-exposure period to the initial PCI opening. Reference communities are those with the same baseline PCI status but did not experience new PCI opening during the study period. Coefficients represent changes in percentage points, and numbers inside brackets represent 95% CI. ** p<0.01 * p<0.05

eTable 7. Sensitivity analysis that separately models the opening of a very low-volume PCI facility and other PCI facilities

	Stable angina		No prior acute heart condition		1 vessel		3+ vessels	
	**Had PCI within 30-min**	**No PCI at baseline**	**Had PCI within 30-min**	**No PCI at baseline**	**Had PCI within 30-min**	**No PCI at baseline**	**Had PCI within 30-min**	**No PCI at baseline**
On and after low-volume PCI (10-50) opening within 30-min drive time	3.24** [2.49,3.99]	3.26 [-0.03,6.56]	0.45 [-0.14,1.03]	-0.64 [-3.24,1.95]	-0.91** [-1.57,-0.26]	1.99 [-0.83,4.82]	0.77** [0.43,1.10]	-1.84** [-3.23,-0.46]
On and after non-low-volume PCI (>=50) opening within 30-min drive time	0.44 [-0.17,1.05]	3.74** [1.33,6.15]	-0.13 [-0.60,0.35]	-0.42 [-2.32,1.48]	0.04 [-0.49,0.56]	2.37* [0.30,4.43]	0.63** [0.36,0.90]	-2.26** [-3.27,-1.25]

Note: Based on linear probability model with community fixed effects. Reference communities are those with the same baseline PCI status but did not experience new PCI opening during the study period. Coefficients represent changes in percentage points, and numbers inside brackets represent 95% CI. ** p<0.01 * p<0.05

© 2026 Shen Y et al. *JAMA Network Open*.

eTable 8. Number of treatment communities and patients by year of exposure to PCI opening

Communities with baseline access within 30 min				
Year of exposure	N of treatment communities	%	N of patients living in treatment communities during study period	%
All	**6,078**		**567,236**	
No exposure	4,907	81%	124,599	22%
2012	414	7%	184,065	32%
2013	106	2%	28,351	5%
2014	176	3%	74,144	13%
2015	254	4%	110,966	20%
2016	71	1%	15,815	3%
2017	34	1%	1,074	0%
2018	10	0%	50	0%
2019	45	1%	16,097	3%
2020	17	0%	1,487	0%
2021	29	0%	9,752	2%
2022	15	0%	836	0%
Communities with no baseline access to PCI within 30 min				
Total patients				
All	**741**		**84,349**	
No exposure	701	95%	66,784	79%
2012	12	2%	9,714	12%

© 2026 Shen Y et al. *JAMA Network Open*.

**2013**	2	0%	202	0%
**2014**	1	0%	145	0%
**2015**	5	1%	2,755	3%
**2016**	0	0%	0	0%
**2017**	9	1%	2,256	3%
**2018**	0	0%	0	0%
**2019**	0	0%	0	0%
**2020**	6	1%	334	0%
**2021**	1	0%	466	1%
**2022**	4	1%	1,693	2%

© 2026 Shen Y et al. *JAMA Network Open*.
