# Supplementary material for: Community-Level Procedure Volume and Patient Health Profiles Following PCI-Capable Facility Openings
Source: JAMA Netw Open. 2026 Mar 30;9(3):e262420. doi: 10.1001/jamanetworkopen.2026.2420 (PMC13036571; doi:10.1001/jamanetworkopen.2026.2420)
Supplement: Supplement 2. — Data Sharing Statement [file jamanetwopen-e262420-s002.pdf]

Data Sharing Statement

Shen. Community-Level Procedure Volume and Patient Health Profiles Following PCI-Capable Facility Openings. *JAMA Netw Open*. Published March 30, 2026. doi:10.1001/jamanetworkopen.2026.2420

Data
Data available: No
